# Hepatocytes: A key role in liver inflammation

**DOI:** 10.3389/fimmu.2022.1083780

**Published:** 2023-01-18

**Authors:** Jin Gong, Wei Tu, Jingmei Liu, Dean Tian

**Affiliations:** Department of Gastroenterology, Tongji Hospital of Tongji Medical College, Huazhong University of Science and Technology, Wuhan, China

**Keywords:** hepatocyte, organelle damage, hepatic inflammation, extracellular vesicles, cytokines

## Abstract

Hepatocytes, the major parenchymal cells in the liver, are responsible for a variety of cellular functions including carbohydrate, lipid and protein metabolism, detoxification and immune cell activation to maintain liver homeotasis. Recent studies show hepatocytes play a pivotal role in liver inflammation. After receiving liver insults and inflammatory signals, hepatocytes may undergo organelle damage, and further respond by releasing mediators and expressing molecules that can act in the microenvironment as well as initiate a robust inflammatory response. In this review, we summarize how the hepatic organelle damage link to liver inflammation and introduce numerous hepatocyte-derived pro-inflammatory factors in response to chronic liver injury.

## 1 Introduction

Chronic liver disease is characterized by hepatocyte injury and inflammation that lead to the development of cirrhosis and liver cancer, accounting for approximately 2 million deaths every year worldwide (1). Multiple etiologies include chronic HBV and HCV infection, nonalcoholic steatohepatitis (NASH), alcoholic liver disease, and autoimmune liver disease cause the global burden of liver disease. Hepatocytes comprise the majority (~85%) of the liver mass, and play a role in various biochemical and metabolic functions (2). Traditional concepts viewed hepatocytes as targets of immune or insults mediated injury, resulting in hepatocyte death which identified as a typical pathological feature in liver disease. However, recent studies have emphasized a role for hepatocyte as active drivers in liver inflammation and fibrosis through intercellular communication (3). Organelle damage, including mitochondria, lysosome, endoplasmic reticulum may determine the severity of hepatocyte injury (4). It is widely accepted that sterile hepatocyte death leads to the release of damage-associated molecular patterns (DAMPs), which are recognized by the innate immune system through pattern recognition receptors, and exaggerate inflammatory response in liver (5). What’s more, stressed hepatocytes engage in liver inflammation as well, for they can change their phenotype, make an adaptation to the microenvironment and alter their surrounding cell populations (2). Substantial evidence show that hepatocytes constitutively produce and secrete a variety of mediators that play important roles in immune regulation and fibrosis (6, 7). In this review, we will provide current literature investigating the adaptive and maladaptive alterations of hepatocytes during the initiation of liver injury, and how the stressed hepatocytes interact with the surrounding cells to trigger a proinflammatory microenvironment in chronic liver disease.

## 2 Endoplasmic reticulum stress in hepatocytes links to liver inflammation

**Figure 1:**
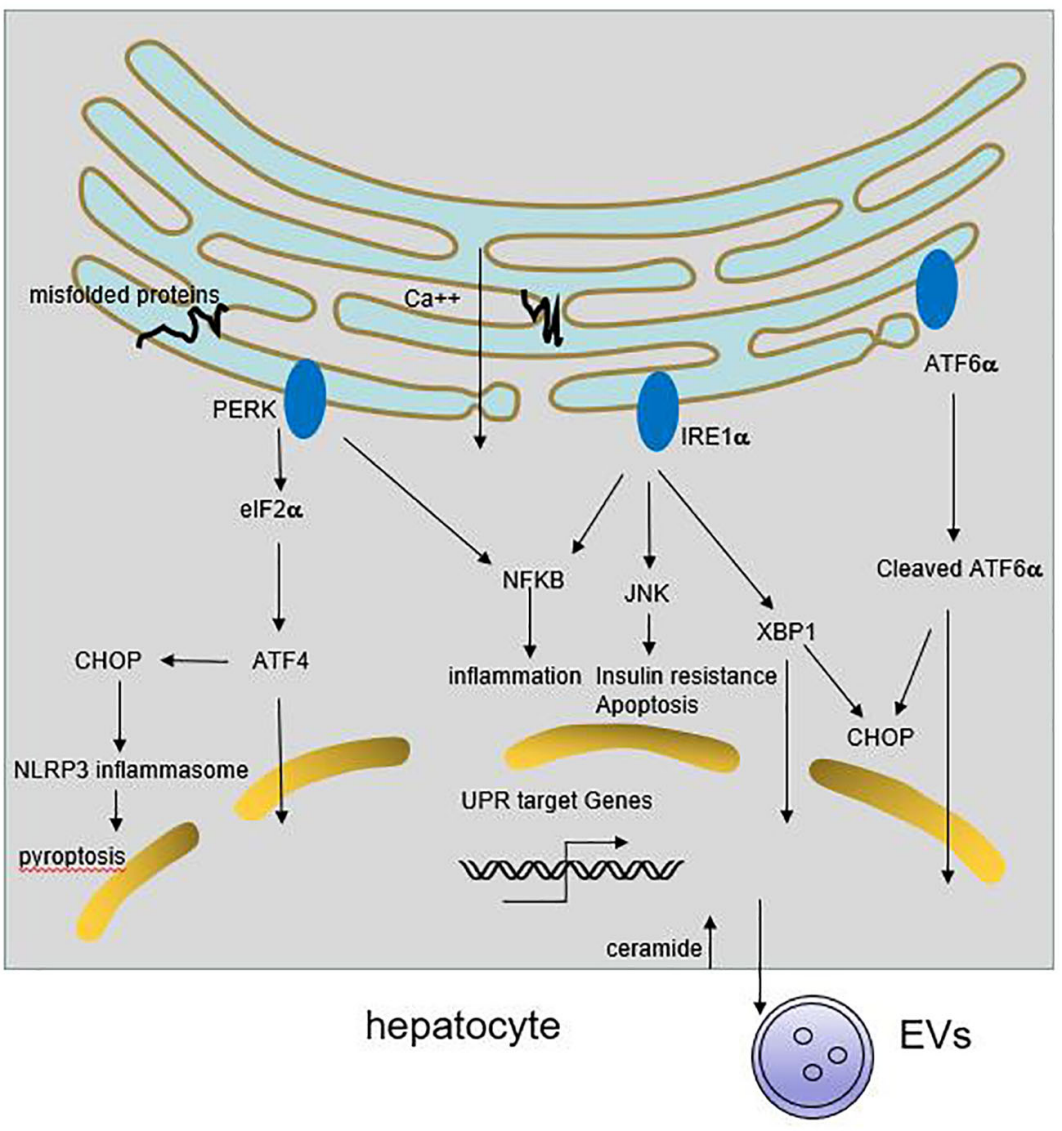
Role of ER stress in liver inflammation. Multiple stimuli lead to the activation of UPR response in hepatocyte. The three ER transmembrane sensors, PERK, IRE1 and ATF6, coordinately through downstream signaling cascades to resolve the protein folding defect and promote cell survival. If the adaptive UPR is overwhelmed by sustained or massive ER stress, it leads to hepatocyte steatosis and death. Meanwhile, ER stress may trigger NFκB and JNKs activation, resulting in release of proinflammatory cytokines. On the other hand, ER stress can induce CHOP-dependent NLRP3 inflammasome activation in hepatocytes. Besides, activation of IRE1A in hepatocytes promotes the release of inflammatory extracellular vesicles (EVs), thereby accummulating immune cells infiltration.

Endoplasmic reticulum (ER) is the major site of secretory and transmembrane protein folding, calcium homeostasis and lipid synthesis. Upon the accumulation of misfolded proteins in the ER, unfolded protein response (UPR) is activated by three ER-transmembrane sensors, namely PKR like ER kinase (PERK), activating transcription factor 6 (ATF6), and inositol requiring enzyme 1 (IRE1), coordinately through downstream factors including X-box binding protein 1 (XBP1), α-subunit of eukaryotic initiation factor 2 (eIF2α), C/EBP homologous protein (CHOP), activating Transcription Factor 4 (ATF4), to resolve the protein folding defect (8). Sustained or massive ER stress leads to hepatocyte steatosis and apoptosis (9) (**Figure 1**).

ER stress is observed in many chronic liver diseases. Chronic ER stress plays a causative role in NAFLD progression by promoting lipogenesis, disturbing mitochondrial function and modulating insulin signaling (10). ER stress markers are shown to decline in livers of obese patients following weight loss after bariatric surgery (11). It has confirmed that impaired autophagic flux is associated with increased ER stress in livers from patients with biopsy-proven NASH during the development of NAFLD (12). Various HBV and HCV proteins localize inside the ER lumen and are undergo envelopment. HBV infection can cause ER stress, which enhance HBV viral replication by initiating autophagy (13). Moreover, chronic HCV infection induce ER stress and the minimal expression of UPR target genes, which confers hepatocytes adaptation and resistance to liver injury (14–16). Hepatic PHLDA3 regulates ER stress-induced hepatocyte death through Akt inhibition in HCV hepatitis (17). Besides, it is reported that interferon regulatory factor 3 (IRF3) is activated by ER stress and induce hepatocyte apoptosis in early alcoholic liver disease (18).

Under chronic ER stress, UPR is linked to the activation of several inflammatory response pathways including NFκB, JNK, ROS, IL-6, TNF-α (8, 19). Activated IRE1α induces JNKs activation, and subsequent implicates in cell pro-inflammatory and pro-apoptotic pathways. Knockdown of JNK1 gene protects mice from the development of obesity and insulin resistance (20). Enhanced ER stress can trigger NFκB activation through IRE1α and PERK pathway, followed by the secretion of inflammatory and chemotactic cytokines in hepatocytes (21, 22). Some HCV and HBV protein accumulate at the ER membranes which cause a deregulation of Ca2+ flux, generation of reactive oxygen and nitrogen species, and the resulting ER stress could induce IL-8 transcription (10, 23, 24). ER stress also induces CHOP-dependent NOD-like receptor family, pyrin domain-containing 3 (NLRP3) inflammasome activation in hepatocytes, potentially causing pyroptotic death and hepatic inflammation in patients with HBV-associated liver failure and NAFLD (25, 26). Recent study shows that activation of IRE1A in hepatocytes promotes the release of inflammatory extracellular vesicles (EVs), which recruit macrophages to liver, resulting in liver inflammation and injury in steatohepatitis (27). Therefore, chronic ER stress cause inflammation and the deregulation of lipid metabolism, that further exacerbate liver diseases.

## 3 Autophagy dysregulation in hepatocytes leads to liver inflammation

Autophagy is a catabolic lysosomal process responsible for clearing damaged proteins, dysfunction organelles and lipid droplets. It is considered as a cellular response to maintain energy balance and in reaction to multiple of cellular stress, such as starvation, hypoxia, and viral infection (28).

Autophagy generally plays a protective role in hepatocytes, since they can protect against steatosis and hepatocyte death. It is reported autophagy can selectively degrades lipid droplets, termed lipophagy, as evidenced by the increase in lipid accumulation upon inhibition of autophagy in hepatocytes (29). Recent studies with specific genetic inhibition of autophagy have established that hepatocytes are more susceptible to various liver injury, such as alcohol, toxic agents, lipotoxic metabolites, and pro-inflammatory factors. Autophagy may promote cell survival by clearing misfolded proteins, lipids and damaged mitochondria (30–33).

Studies show that regulation of autophagy links to the progression of chronic liver diseases. Impaired autophagic flux links to steatosis and progression to NASH in NAFLD patients and mouse models by genetic or phamacological inhibition of autophagy (12). Shen et al. have uncovered pathogenesis of IL-1β-induced liver injury in steatohepatitis by finding that IL-1β becomes cytotoxic and pro-inflammatory to hepatocytes when inhibition of autophagy, leading to cell necrosis and liver inflammation (34). Although autophagy can alleviate hepatocyte apoptosis and steatosis in acute alcohol liver disease (35), decrease autophagic flux in hepatocyte is observed in models of chronic alcohol exposure (36, 37). A significant decrease in UQCRC2 protein expression cause impaired mitophagy, which may aggravate MLKL-mediated hepatocyte necroptosis and inflammation in alcoholic liver disease (38, 39). Furthermore, early autophagy enhance HBV infection and envelopment (40). Inhibition of autophagy by liver-specific knockout of Atg5 in HBV transgenic mice can obviously reduce HBV DNA level (41). Additionally, autophagy plays an important role in HBV-mediated immune response (40). GAL9, a type I IFN-stimulated gene, exerts effect on direct autophagic degradation of HBc in HBV-infected hepatocytes (42). ATG12 is required for HBV replication and impediment of the IFN signaling pathway, as evidence by decreased levels of IFN-α, IFN-β in ATG12-knockdown hepatocytes (43). Autophagy inhibition also abrogates HBx-induced activation of nuclear factor-κB (NF-κB) and production of interleukin-6 (IL-6), IL-8, and CXCL2 (44). Similarly, autophagy is required to promote HCV replication, partly through suppression of innate immunity (45, 46). HCV-induced autophagy can suppress host innate immune response through autophagic degradation of TRAF6, which is an important signaling molecule that mediates the activation of NF-kB and expression of cytokines and interferons (47). Meanwhile, loss of autophagy signaling upregulates HCV-induced cytoplasmic RIG-I signaling and IFN-β–mediated antiviral responses (48). Interference of HCV-induced mitophagy by Drp1 silencing enhances innate immune signaling (49). The correlation between AIH and autophagy in hepatocyte is not clear. It has been observed increased LC3 and p62 expression in hepatocytes of AIH patients, and p62 level is strongly correlated with necroinflammatory grade, which indicates that decreasing autophagic activity may be linked to severity of inflammation in AIH (50).

## 4 The role of hepatic mitochondrial dysfunction in liver inflammation

**Figure 2:**
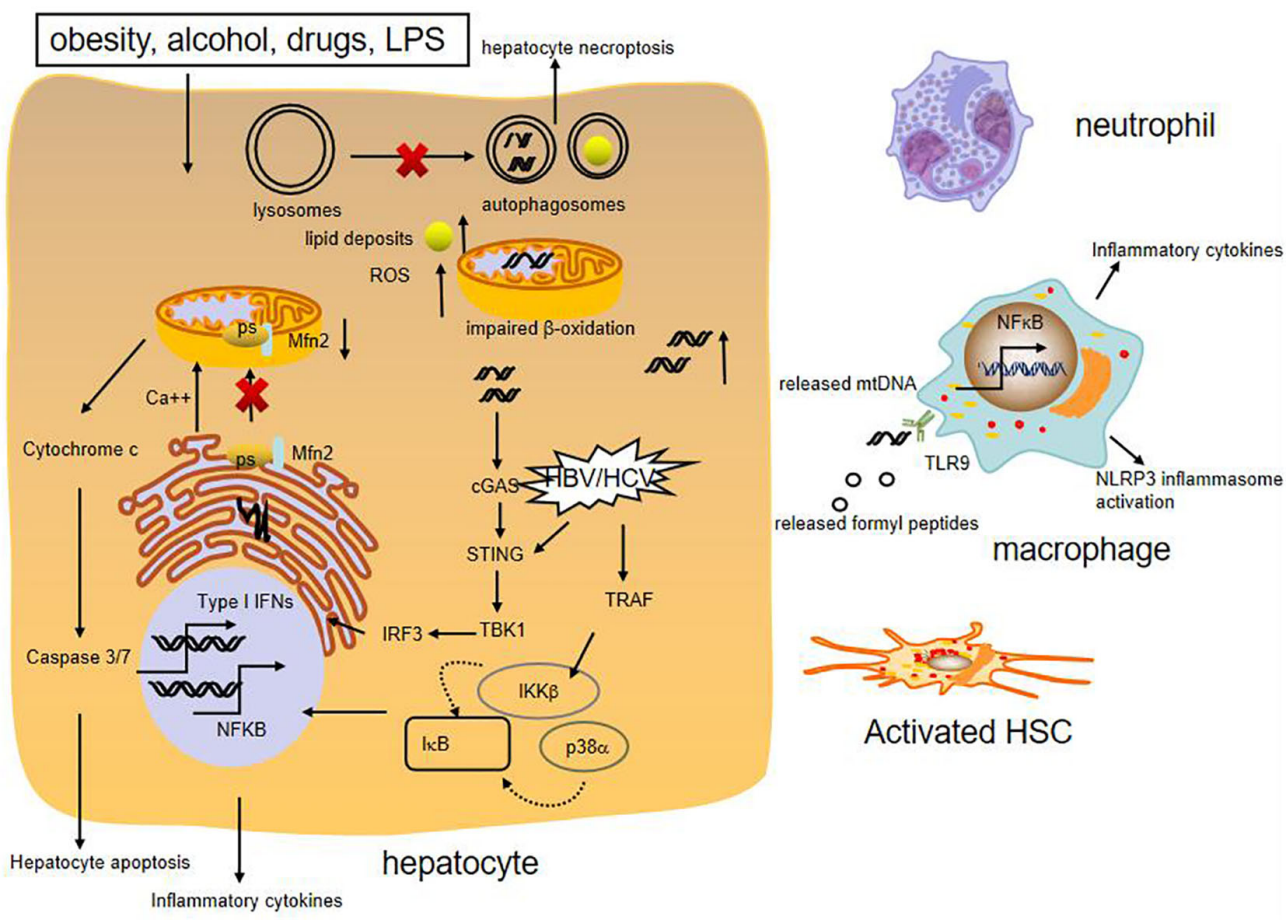
Role of mitochondrial damage in liver inflammation. Various liver injury impair mitochondrial respiration and increase ROS formation, cause mtDNA damage. High levels of ROS can increase synthesis of cytokines, which cause apoptosis and necrosis of hepatocytes. The presence of mtDNA in the cytosol or circulation can trigger proinflammatory and type I IFN responses. Moreover, release of mitochondria-derived danger signals, such as mtDNA, formylated proteins, can attract macrophage and neutrohphils, resulting in activation of NFκB and NLRP3 inflammasome. MtDNA also promotes fibrogenic activation of HSCs. Besides, reduced expression of mitochondrial protein Mfn2 leads to deficient ER-mitochondrial phosphatidylserine transfer, which provokes liver inflammation. HBV and HCV can activate innate immune antiviral signaling and inflammatory pathways through induction of type I interferons and expression of inflammatory cytokines by NFκB.

Mitochondria are abundant in the liver and required for lipid metabolism and energy production. They can directly or indirectly influence other cellular components such as the lysosomes, the endoplasmic reticulum (ER), and cytosolic pathway, to meet the cellular demands and alleviate mitochondrial dysfunction (51). Generally, mitochondria maintain normal morphology and homeostasis by the way of mitochondrial quality control, including the regulation of mitochondrial fusion, fission, biogenesis, and mitophagy (52). When they fail to adapt to various stress, they can release mitochondrial DNA (mtDNA) in the cytosol or circulation, which could induce cGAS-STING-dependent type I interferon (IFN) response. Furthermore, mtDNA synthesis can activate the NLRP3 inflammasome which initiates inflammation (53). In addition, mitochondrial dysfunction can generate excessive reactive oxygen species (ROS), which stimulate synthesis of cytokines to amplify the inflammatory cascade reaction and cause apoptosis and necrosis of hepatocytes (52) (**Figure 2**).

Emerging evidence shows that mitochondria dysfunction, especially mitochondria-derived immunogenic components (including its DNA) have profound impacts on the development of various chronic liver diseases. It is reported that NASH patients produce high mitochondrial levels of ROS and ROS-mediated mtDNA damage (54). Moreover, mtDNA is elevated in the serum of NASH patients and in association with histological degree of hepatic fibrosis. The mtDNA released from injured hepatocyte mitochondria could directly activate hepatic stellate cells (HSCs) and promote inflammation through binding to endosomal TLR9 of Kupffer cells (55, 56). Besides, Mitochondrial protein mitofusion 2 (Mfn2) plays an important role in connecting ER membranes to mitochondria and mitochondrial fusion, studies show that hepatic mfn2 deficiency impairs ER-mitochondrial phosphatidylserine transfer and mitochondrial function, leading to ER stress and liver inflammation in NAFLD (57, 58). Mitochondrial dysregulation is also observed in hepatocytes of patients with AIH and experimental mouse model with immune-mediated liver injury. Blockade of dynamin-related protein 1(Drp1)-mediated mitochondrial fission protects mice from concanavalin A (ConA)-induced liver injury (59). In addition, hepatic ATF4 plays a pathological role in alcohol-induced mitochondrial dysfunction and liver injury by repressing TFAM expression, while AMPK protects against alcohol-induced liver injury through up-regulating mitophagy (39, 60). Apart from the above, chronic HBV and HCV infection could induce mitochondrial oxidative stress and mitochondrial antiviral signaling-mediated innate immune signaling as well (61, 62).

## 5 Mediators involved in intercellular communication

During chronic liver injury, stressed hepatocytes can release mediators that involved in crosstalk between hepatocytes and surrounding cell populations. Besides, hepatocytes serve as liver-resident nonprofessional antigen presenting cells (APCs), resulting in a bias toward immune tolerance.

### 5.1 Hepatocyte-derived extracellular vesicles in liver inflammation

Extracellular vesicles (EVs) are homogeneous vesicles containing lipid, nuclear acid, proteins, which can be secreted by various cell types to the extracellular space and circulation. EVs include microvesicles, exosomes and apoptotic bodies depending on their source and molecular structure.

A growing body of evidence have identified EVs as a conveyor mediating intercellular communication in liver diseases (63) (List in **Table 1**). Hepatocyte-derived EVs as pathogenic mediators play a role in NASH (77). Hepatocyte-derived exosomes from early onset obese mice promote insulin sensitivity through miR-3075 (64). The increase in plasma mtDNA contained in EVs of hepatocyte origin could drive NASH development by activation of TLR9 (56). EVs are also shown as mediators of toxic lipid-induced intercellular signaling. Lipotoxic activation of hepatocytes induce release of EVs enriched in ceramide, CXCL10, miR-192-5p, which trigger chemotaxis and inflammatory phenotype switch of macrophages (65–68). Besides, EVs mediate cell-to-cell communication in alcoholic liver disease. In patients with alcoholic hepatitis, the number of circulating EVs is reported higher than those in healthy individuals, and the EVs contain elevated levels of miR­122, miR­192 and miR­309 (69). Hepatocyte-derived EVs modulate activation of liver marcophages by transferring miRNA-122 and CD40­ligand after alcohol exposure (70, 71). In addition, it is reported that exosomes isolated from sera of chronic HBV and HCV infected patients or supernatants of those hepatocytes contain viral RNA, which can mediate viral transmission to naive hepatocytes (72, 75). These hepatic derived-exosomes involve in host innate immune response and virus-mediated immunosuppression. HCV-associated exosomes can transfer immunomodulatory viral RNA from infected cells to neighboring immune cells and trigger myeloid-derived suppressor cell expansion (73). EVs from hepatitis C virus-infected cells stimulate monocytes to produce galectin-9, which induces apoptosis of hepatitis C virus-specific T cells and increases inhibitory regulatory T cells (74). Similarly, HBV components are observed to be transmitted into NK cells by exosomes, resulting in NK-cell dysfunction (75). Exosomes also can regulate innate immune response against HBV through inducing NKG2D ligand expression in macrophages, which stimulates IFN-γ from NK cells, and suppressing IL-12p35 mRNA expression to counteract he host innate immune response (76). In a word, EVs exert a crucial role on the crosstalk between hepatocytes and nonparenchymal liver cells.

**Table 1:** Biosynthesis of secreted extracellular vesicles by hepatocytes.

Molecules	role	liver disease model	references
miR-3075	promote insulin sensitivity, promote proinflammatory activation of macrophages	a HFD diet induced-obesity model	(64)
mtDNA	activate TLR9 on Kupffer cells	Experimental NASH model induced by HFD diet	(56)
ceramides	activate macrophage chemotaxis	hepatocytes treated with palmitate, a HFD diet model with hepatocyte-specific disruption of Ire1a	(27, 65)
TRAIL	activate an inflammatory phenotype in macrophages	hepatocytes treated with palmitate, Experimental NASH model induced by HFD diet	(66)
CXCL10	induce macrophage chemotaxis	hepatocytes treated with palmitate or LPC, a FFC diet-fed Mlk3 deficient mice	(67)
miR-192-5p	activate an inflammatory phenotype in macrophages	Experimental NASH model induced by high-fat high-cholesterol diet	(68)
miR122	activate an inflammatory phenotype in macrophages, potential diagnostic markers	patients with alcoholic hepatitis Experimental AH model induced by alcohol-fed mice	(69, 70)
miR192	potential diagnostic markers	patients with alcoholic hepatitis Experimental AH model induced by alcohol-fed mice	(69)
miR309	potential diagnostic markers	patients with alcoholic hepatitis Experimental AH model induced by alcohol-fed mice	(69)
CD40ligand	activate an inflammatory phenotype in macrophages	Experimental AH model induced by alcohol-fed mice	(71)
HCV RNA	mediate viral transmission to naive hepatocytes, transfer immunomodulatory viral RNA to neighboring immune cells, trigger myeloid- derived suppressor cell expansion, induce apoptosis of hepatitis C virus-specific T cells,	hepatitis C virus-infected hepatocytes chronic HCV infected patients	(72–74)
HBV nucleic acids and proteins	induce active infection in naive human hepatocytes, transmit into NK cells and lead to NK-cell dysfunction, stimulate IFN-γ from NK cells and suppress IL-12p35 mRNA expression, transfer of antiviral molecules from liver nonparenchymal cells to hepatocytes	hepatitis B virus-infected hepatocytes chronic HBV infected patients	(75, 76)

HFD, high fat diet; LPC, lysophosphatidylcholine; FFC, fat, fructose and cholesterol.

### 5.2 Hepatic cytokines involved in liver inflammation

Hepatocytes can produce diverse cytokines to regulate liver injury, repair, and inflammation in liver injury. Here, we make a summary of cytokines that involved in the pathogenesis of chronic liver diseases below.

IL-6 can be synthesized by hepatocytes in response to specific stimuli to induce acute phase response, it implicates in the liver regeneration following partial hepatectomy and exerts antiviral effects on limiting the replication of HBV in hepatocytes (78, 79). Moreover, substantial studies show that IL-6 trans-signaling promotes inflammation in chronic liver diseases (80). Excessive lipid accumulation in hepatocytes stimulates IL11 protein secretion, autocrine IL11 activity drives lipotoxicity and underlies the transition from NAFLD to NASH (81). Interleukin 33 (IL-33) functions as an “alarmin” released from hepatocytes in response to tissue damages. It exerts protective effects on hepatocytes through the activation of autophagy and suppression of cell death, meanwhile, it regulates host innate immunity by recruitment and activation of ST2-positive target immune cells in the liver (82). Furthermore, it is responsible for repressing viral transcription, protein production and genome replication in HBV-infected hepatocytes (83). IL-32 is markedly induced in hepatocytes in various liver diseases. It plays an important role in inflammatory response by promoting proinflammatory cytokines such as IL-1β and tumor necrosis factor alpha (TNF-α) (84, 85). IL32 also has a critical role in the pathogenesis of NAFLD, partly due to its association with hepatocyte insulin resistance and cholesterol homeostasis (86, 87). Besides, it can suppress HBV transcription and replication (88). Hepatocyte also can produce several chemokines to attract immune cells in response to liver injury. For example, hepatocyte can express chemokine MCP-1, which recruits macrophages to promote liver steatosis and inflammation in alcoholic and non-alcoholic fatty liver disease. Moreover, hepatic MCP-1 expression is found to regulate fatty acid oxidation resulting in steatosis during chronic alcohol exposure (89, 90). Apart from the above, hepatocytes can secrete high amounts of CXCL1, leading to hepatic neutrophil infiltration through TLR2 and TLR9-dependent pathway in alcohol-mediated liver injury (91). Hepatocyte is the main source for necrotic cell-induced CXCL1 production, which dependent of NF-κB activation by Kupffer cells, resulting in neutrophils mobilization and finally clearing dead cells (92). Another study shows that hepatocyte-specific gp130 signaling is sufficient to induce CXCL1 expression, independent of NF-κB activation, triggering a robust systemic innate immune response (93). Steatotic hepatocytes also can stimulate IL-8 production, an active neutrophil chemoattractant, potentially contributing to hepatic inflammation (94).

### 5.3 Role of hepatocytes in antigen-presentation

In clinical hepatitis, viral or autoimmune especially, hepatocytes can directly modulate immune cells *via* cell-cell interactions. Hepatocytes could function as nonprofessional APCs because they express MHC class II during inflammation. MHC-II overexpressing hepatocytes are capable of activating CD4+ T-cells *in vitro*, but they only induce T helper cell (Th) 2 differentiation, which impair antiviral CD8 T-cell responses and viral clearance (95, 96). Hepatocytes appear to play a role in the liver tolerogenic effect. They can activate CD8+ T cells in a manner that leads to apoptosis of these cells since lack of either costimulatory signals or CD4+ T cell help (97). What’s more, the hepatocytes may endocytose and kill CD8+ T cells that recognize them, a process known as suicidal emperipolesis (98). In viral infection, virus-positive hepatocytes can be eliminated by activated circulating CD8+ T-cells through directly recognizing antigen on hepatocytes, leading to CD8+T-cell exhaustion (99). Among the underlying mechanism, Notch signaling may performed an important regulatory role in the interaction between hepatocytes and T cells activation. It is reported hepatocytes fine -tune liver inflammation by upregulation of Jagged1 and activation of Notch signaling in Th1 cells, resulting in induction of IL10-producing CD4+ T cells (100). Besides, Notch signaling contributes to liver inflammation by regulation of interleukin-22-producing cells in hepatitis B virus infection (101). In addition, hepatocytes may induce tolerance *via* Notch-mediated conversion of CD4(+) T cells into Foxp3(+) Tregs upon TCR stimulation (102). Apart from these, intercellular adhesion molecule 1 (ICAM-1) is involved in CD4+ T cell engulfment by hepatocytes and huh-7 cells by facilitating T cell early adhesion and internalization (103).

## 6 Conclusion

A growing number of evidences have demonstrated stressed hepatocytes exert a pivotal role on the development of inflammation and fibrosis *via* cell-cell interactions during liver injury. In this review, we summarize the role of hepatic organelle disorders in the pathogenesis of chronic liver diseases, especially, their links to liver inflammation. Furthermore, we introduce a wide variety of pro-inflammatory signals carried by hepatocyte derived-EVs that can deliver the message to neighbor target cells and in the circulation to modulate immune response. Besides, we conclude several cytokines and chemokines of hepatocyte origin which engage in chronic liver diseases. Finally, we address briefly antigen-presentation properties of hepatocytes in immune regulation. Understanding of the molecular mechanisms involved in the regulation of hepatic organelle damage, as well as role of hepatocyte in immune regulation may provide us novel insights of dysregulated inflammation during liver injury and identify new therapeutic targets for various liver diseases.

## Author contributions

JG and JL contributed to select the topic of the manuscript. WT collected relevant literature. JG wrote the manuscript. JL and DT reviewed and edited the final version of manuscript. All authors contributed to the article and approved the submitted version.
